# Parental anxiety levels before and after pediatric cardiology evaluation with echocardiography in asymptomatic children with heart murmurs: a prospective study

**DOI:** 10.3389/fped.2026.1901573

**Published:** 2026-07-09

**Authors:** Hikmet Kıztanır, Beyza Elmalı

**Affiliations:** 1 Division of Pediatric Cardiology, Department of Pediatrics, Recep Tayyip Erdogan University Faculty of Medicine, Rize, Türkiye; 2 Faculty of Medicine, Recep Tayyip Erdogan University, Rize, Türkiye

**Keywords:** echocardiography, heart murmur, parental anxiety, pediatric cardiology, STAI-S

## Abstract

**Background:**

Cardiac murmurs are common in childhood, yet their detection often triggers significant parental anxiety. This study aimed to measure anxiety levels in parents of infants and young children under 24 months of age referred to pediatric cardiology due to heart murmurs, using the State-Trait Anxiety Inventory (STAI-S) before and after examination, and to investigate the impact of expert assessment and echocardiography (ECHO).

**Methods:**

This prospective study included parents of 241 asymptomatic children (under 24 months) referred for initial cardiac evaluation due to a murmur. Parental anxiety was measured using the STAI-S before and after a comprehensive clinical evaluation, including ECHO. Participants were categorized based on ECHO findings: normal, benign physiological findings, and minor structural anomalies requiring follow-up.

**Results:**

The mean pre-consultation STAI-S was 51.17 ± 6.34, which significantly decreased to 45.89 ± 7.84 following evaluation [*t* (240) = 10.44, *p* < 0.001, Cohen's *d* = 0.67]. While anxiety levels dropped significantly across all ECHO groups (*p* < 0.001), no statistical difference was found between groups regarding anxiety reduction (*p* = 0.108). Exploratory item analysis revealed that consultation primarily reduced negative emotions rather than immediately increasing positive emotional states. Regression analysis showed that a family history of heart disease was the only significant independent predictor increasing pre-examination anxiety (*β* = 2.129; *p* = 0.031).

**Conclusions:**

Specialized pediatric cardiology consultation and echocardiographic evaluation substantially reduce parental anxiety, independent of socioeconomic characteristics or specific cardiac findings in this cohort. Clinicians should prioritize early, tailored communication for high-risk families with a family history of heart disease to effectively manage the psychological burden of a heart murmur referral.

## Introduction

Cardiac murmurs are a common physical examination finding in childhood and the neonatal period and are the most frequent reason for referrals to pediatric cardiology clinics (1, 2). Studies show that a heart murmur can be detected in approximately 50% to 80% of children at some point in their lives, but the vast majority of these murmurs are innocent or functional murmurs not associated with a structural or functional cardiac abnormality in the heart (3–6). Although pathological murmurs indicative of structural heart disease account for less than 1% of all cases, it may not always be possible for physicians to differentiate between benign and pathological murmurs solely through physical examination (3, 7). This situation leads to asymptomatic children often being referred to a pediatric cardiologist for further evaluation to rule out serious heart disease (1, 2). Notifying a family that a heart murmur has been detected in their child, and the subsequent referral process, can cause significant anxiety and worry for parents. Parents often tend to associate the term “murmur” with serious heart disease, the need for surgery, or a fear of severe cardiac complications, which can lead to misconceptions, even to the point of restricting their children's physical activities (5, 7, 8). Even if the child is asymptomatic or the referring physician suggests that the murmur is likely benign, this is not always enough to reduce the perceived threat level of the parents, and this period of uncertainty can negatively affect the parents' overall well-being (1, 2, 5). Even when a specialist diagnoses an innocent heart murmur, it has been observed that some parents still harbor concerns that their child has heart disease (5, 8, 9). A detailed physical examination, and especially echocardiographic evaluation, performed by a pediatric cardiologist is the most reliable method to eliminate diagnostic uncertainty, rule out structural heart diseases, and reassure the family (9–11). Studies in the literature show that after expert consultation, echocardiography (ECHO), and detailed information, parents experience a significant decrease in anxiety levels and an increase in their understanding of the disease (9, 10). However, while debates continue regarding the routine use and cost-effectiveness of ECHO, its role in alleviating parental anxiety is well recognized (3, 10). The aim of this study was to measure the anxiety levels of parents of infants and young children under 24 months of age referred to pediatric cardiology due to heart murmurs, using the State-Trait Anxiety Inventory-State (STAI-S) questionnaire before and after pediatric cardiology evaluation, and to investigate the effect of expert evaluation on parents' anxiety levels.

## Materials and methods

### Study design and participants

This prospective study included children under 24 months of age (and one consenting parent) referred to our pediatric cardiology clinic for their first evaluation of an asymptomatic heart murmur. Patients were prospectively enrolled during outpatient visits. According to national healthcare regulations, admission required a formal referral from a pediatrician and a central registry appointment, which typically resulted in a 1-to-2-week waiting period. At our institution, all such patients routinely undergo ECHO to definitively rule out structural anomalies and provide objective reassurance to families. Those caring for infants with chromosomal or congenital anomalies, suspicious findings on prenatal ultrasound, or symptoms suggestive of underlying cardiovascular or respiratory disorders were excluded from the study. All infants were clinically stable at the time of examination. The study was approved by our hospital's clinical research ethics committee with decision number 2024/311 dated 26 December 2024.

### Data collection and measures

Parents (either the mother or the father) of children found to have heart murmurs during examinations were administered the State Anxiety Scale of the State-Trait Anxiety Inventory (STAI-S); only one parent per child was enrolled in the study based on who accompanied the child during the outpatient visit and voluntarily agreed to complete the questionnaires. The trait anxiety scale was not utilized in this study as the primary aim was to capture immediate situational distress. Before the examination, the responding parents provided information regarding their child's age, parental education level, family history of heart disease or cardiac murmur, number of children in the family, the patient's birth order (first, second, third), and whether the child had any complaints at the time the murmur was detected or at presentation to the pediatric cardiology outpatient clinic.

The initial pre-consultation STAI-S questionnaire was completed by the parents in the waiting area, just prior to entering the examination room. The comprehensive clinical evaluation, including the physical examination and echocardiographic imaging, lasted approximately 10–15 min across all patients. Immediately following the examination, the findings were thoroughly explained to the parents, a written report was provided, and a follow-up appointment was scheduled if necessary. Subsequently, to ensure a standardized recovery window, parents completed the post-consultation STAI-S questionnaire approximately 10–15 min after the conclusion of this clinical interview. The total interval between the pre- and post-consultation assessments averaged approximately 20–30 min, and this timeline did not differ significantly between the study groups.

The STAI-S is a widely used self-administered questionnaire that measures situational anxiety levels, assessing how an individual feels at a particular moment and under specific circumstances. Its Turkish translation, cultural adaptation, and validation were previously established by Öner and Le Compte (12). The STAI-S consists of 20 items, with total scores ranging from 20 to 80; higher scores indicate greater anxiety, and a score of 40 or higher is generally considered to reflect significant anxiety (12, 13). The total STAI-S score is calculated using a specific standard scoring procedure (50 + Direct-Reverse), where ‘Direct’ represents the sum of raw scores from items reflecting negative emotions (10 items), and ‘Reverse’ represents the sum of items reflecting positive emotions (10 items) (12). In our study, the total STAI-S score was calculated using the standard 20-item questionnaire. To confirm the reliability of this full instrument specifically within our participant population, we calculated its internal consistency. The Cronbach's alpha for the full 20-item scale in our cohort was 0.682 pre-consultation and 0.812 post-consultation, indicating acceptable to good reliability. However, for the item-level exploratory analysis (Table 1), we specifically extracted and analyzed a well-validated 6-item subset (comprising 3 direct and 3 reverse items: tension, upset, worry, relaxation, contentment, and calmness). These specific 6 items were chosen because they constitute the established short-form of the STAI-S developed by Marteau and Bekker (14) As demonstrated in the literature, this 6-item subset shows high internal consistency (Cronbach's alpha 0.81–0.85) and an excellent correlation (*r* > 0.93) with the full 20-item scale, making it a highly reliable and representative surrogate for capturing acute emotional shifts in perinatal and pediatric settings (9, 15) Due to strict copyright protections of the STAI instrument (Mind Garden, Inc.), the exact phrasing of the items cannot be reproduced, and providing the full 20-item list as supplementary material is restricted; thus, these 6 core items are represented solely by their underlying emotional constructs.

**Table 1:** Comparison of sTAI-S Sub-item scores before and after consultation across ECHO groups.

Item	**Normal (Pre)**	**Normal (Post)**	**P**	**Benign physiological findings (Pre)**	**Benign physiological findings (Post)**	**p**	**Minor structural heart anomalies requiring follow-up (Pre)**	**Minor structural heart anomalies requiring follow-up (Post)**	**p**
Item 1: Feeling of relaxation (R)	1.82 ± 0.56	1.85 ± 0.74	0.742	2.03 ± 0.78	1.95 ± 0.75	0.294	2.06 ± 0.81	2.16 ± 0.86	0.557
Item 2: Feeling of contentment (R)	1.87 ± 0.74	1.81 ± 0.76	0.59	2.07 ± 0.83	1.95 ± 0.81	0.115	2.06 ± 0.63	2.03 ± 0.80	0.845
Item 3: Feeling of calmness (R)	1.90 ± 0.67	1.74 ± 0.68	0.124	2.08 ± 0.80	1.89 ± 0.83	**0.014**	1.90 ± 0.65	1.87 ± 0.72	0.839
Item 4: Feeling of tension (D)	2.06 ± 0.92	1.50 ± 0.80	**<0.001**	2.04 ± 0.86	1.44 ± 0.72	**< 0.001**	2.19 ± 1.05	1.68 ± 1.05	**0**.**043**
Item 5: Feeling upset (D)	2.24 ± 0.94	1.58 ± 0.82	**<0.001**	2.30 ± 0.85	1.58 ± 0.87	**< 0.001**	2.55 ± 0.68	1.58 ± 1.03	**< 0.001**
Item 6: Feeling of worry (D)	2.06 ± 0.92	1.50 ± 0.82	**<0.001**	2.05 ± 0.90	1.45 ± 0.79	**< 0.001**	2.32 ± 1.01	1.65 ± 0.98	**0**.**001**

Data are presented as Mean ± Standard Deviation. Within-group comparisons were performed using the paired Student's *t*-test. R, Reverse-scored items (representing positive emotions). D, Direct-scored items (representing negative emotions), Values in bold indicate statistically significant differences (*p* < 0.05). Note: Exact item wordings are strictly copyrighted by Mind Garden, Inc. and are represented here by their underlying emotional constructs.

### Echocardiographic classification criteria

Based on the comprehensive ECHO evaluations, patient data were classified into three distinct diagnostic groups based on anatomical severity and follow-up requirements:

Normal Group: Infants with completely normal cardiac anatomy and no detectable structural variations.

Benign Physiological Findings Group: This category included hemodynamically insignificant, transient, or normal variant findings that do not restrict childhood activities, specifically: Patent Foramen Ovale (PFO), tiny Patent Ductus Arteriosus (PDA; defined strictly as a ductal diameter <1.5 mm with no left-sided heart chambers enlargement), and mild Peripheral Pulmonary Stenosis (PPS; exhibiting physiological resolution).

Minor Structural Heart Anomalies Group: Children with structural defects that require routine non-invasive clinical follow-up but no immediate medical or surgical intervention, including Atrial Septal Defect (ASD), Ventricular Septal Defect (VSD), Bicuspid Aortic Valve (BAV), and Mitral Regurgitation (MR).

### Statistical analysis

Statistical analysis was performed using IBM SPSS software version 25.0 (IBM Corp., Armonk, NY). Survey scores are presented as mean ± standard deviation. A paired Student's *t*-test was utilized to compare STAI-S scores before and after cardiological consultation. Within-group comparisons (i.e., before-after consultation) were performed using paired Student's *t*-test, while between-group comparisons were made using one-way ANOVA with Tukey test for multiple comparisons. Multivariate linear regression analysis was applied to investigate the role of various parameters on STAI-S scores.

## Results

A total of 241 children and their parents were enrolled in the study from September 2025 to January 2026. The median age of the children at the time of pediatric cardiology evaluation was 3 months [interquartile range (IQR): 1–6 months], indicating that the study population consisted predominantly of newborns and young infants. Female infants had a median age of 2 months (IQR: 1–6 months), whereas male infants had a median age of 3 months (IQR: 2–6.25 months). The detailed demographic and clinical characteristics of the study population, including child's gender, presence of complaints, and birth order, are presented in Table 2.

**Table 2:** Demographic and clinical characteristics of the participants (*N* = 241).

**Characteristics**	***n* (%) or Median (Min–Max)**
Age (Years)	31 (18–51)
Gender	
Female (Mother)	154 (63.9%)
Male (Father)	87 (36.1%)
Child age at evaluation, months, median (IQR)	3 (1–6)
Female sex, *n* (%)	117 (48.5%)
Male sex, *n* (%)	124 (51.5%)
Education Level	
University	115 (47.7%)
High School	96 (39.8%)
Primary Education	30 (12.4%)
Monthly Income Level	
0–30.000 TL	82 (34.0%)
30.001–50.000 TL	66 (27.4%)
50.001–75.000 TL	58 (24.1%)
75.001-100.000 TL	17 (7.1%)
100.001-150.000 TL	13 (5.4%)
150.001 TL and above	5 (2.1%)
Total Number of Children in the Family	
One child	127 (52.7%)
Two children	73 (30.3%)
Three children	27 (11.2%)
Four or more	14 (5.8%)
Family History of Heart Disease	
Yes	55 (22.8%)
No	186 (77.2%)
Echocardiography Groups	
Normal	62 (25.7%)
Benign physiological findings^a^	148 (61.4%)
Minor structural heart anomalies requiring follow-up^b^	31 (12.9%)

a Benign physiological findings: PFO, Patent Foramen Ovale; PDA, tiny Patent Ductus Arteriosus, PPS, mild Peripheral Pulmonary Stenosis.

b Minor structural heart anomalies requiring follow-up: ASD, Atrial Septal Defect; VSD, Ventricular Septal Defect; BAV, Bicuspid Aortic Valve; MR, Mitral Regurgitation.

According to the ECHO findings, 25.7% (*n* = 62) of the children were normal, 61.4% (*n* = 148) had benign physiological findings (130 with Patent Foramen Ovale, 7 with tiny Patent Ductus Arteriosus, 11 with Peripheral Pulmonary Stenosis), and 12.9% (*n* = 31) had minor structural heart anomalies requiring follow-up (22 with Atrial Septal Defect, 5 with Ventricular Septal Defect, 1 with Bicuspid Aortic Valve, 3 with Mitral Regurgitation). The most frequently detected ECHO finding was Patent Foramen Ovale (PFO) at 53.9% (*n* = 130).

Comparison of parental situational anxiety levels before and after cardiology examination showed that the mean total anxiety score decreased significantly from 51.17 ± 6.34 to 45.89 ± 7.84 [*t* (240) = 10.44; *p* < 0.001]. This change represents a moderate-to-large effect size (Cohen's *d* = 0.67). Notably, the reduction was particularly pronounced in items directly related to anxiety (Direct items; *d* = 0.92), indicating a significant decrease in the negative emotional burden on parents after the examination. The results are presented in Table 3.

**Table 3:** STAI-S questionnaire results before and after cardiology consultation.

**Variable**	**Pre-test (Mean ± SD)**	**Post-test (Mean ± SD)**	**Difference**	**t**	**df**	**p**	**Cohen's d (Effect Size)**
Total Anxiety Score	51.17 ± 6.34	45.89 ± 7.84	−5.28	10.440	240	**< 0.001**	0.672
Direct Items	21.25 ± 5.19	15.11 ± 6.49	−6.14	14.406	240	**< 0.001**	0.928
Reverse Items	20.08 ± 5.00	19.22 ± 5.70	−0.86	2.613	240	**0.010**	0.168

SD, Standard Deviation; df, Degrees of Freedom (*n* − 1), Values in bold indicate statistically significant differences (*p* < 0.05). Cohen's d criteria, 0.2 = small, 0.5 = medium, 0.8 = large; Direct (D), Items scored directly (reflecting negative emotions); Reverse (R), Items scored in reverse (reflecting positive emotions).

When anxiety scores were analyzed according to parent gender, both mothers and fathers demonstrated significant reductions in STAI-S scores following pediatric cardiology consultation and echocardiographic evaluation. Mothers showed a decrease from 50.45 ± 6.65 to 45.48 ± 8.61 (*t* = 7.12, *p* < 0.001; Cohen's d = 0.57), whereas fathers showed a decrease from 52.44 ± 5.56 to 46.62 ± 6.21 (*t* = 9.14, *p* < 0.001; Cohen's *d* = 0.98). Fathers had slightly higher baseline anxiety scores than mothers and demonstrated a larger reduction in anxiety after consultation. The detailed results are presented in Table 4.

**Table 4:** Comparison of sTAI-S scores between mothers and fathers before and after pediatric cardiology consultation.

Parent	***n***	**Pre-test (Mean ± SD)**	**Post-test (Mean ± SD)**	***t***	***p***	**Cohen's d (Effect Size)**
Mothers	154	50.45 ± 6.65	45.48 ± 8.61	7.12	**<0.001**	0.57 (Medium)
Fathers	87	52.44 ± 5.56	46.62 ± 6.21	9.14	**<0.001**	0.98 (Large)

STAI-S, State-Trait Anxiety Inventory-State Form; SD, Standard Deviation. Within-group comparisons were performed using paired Student's *t*-test. Values in bold indicate statistically significant differences (*p* < 0.05).

According to the ECHO findings, parental anxiety was statistically significantly reduced (*p* < 0.001) in all groups (normal, benign physiological findings, minor structural heart anomalies requiring follow-up), the detailed results are presented in Table 5. No statistically significant difference was found in the comparison of the amount of decrease in anxiety scores of the groups (ANOVA) [F (2, 238) = 2.24; *p* = 0.108].

**Table 5:** STAI-S questionnaire results based on ECHO findings and their impact on parental anxiety.

ECHO Result Group	***n***	**Pre-test (Mean ± SD)**	**Post-test (Mean ± SD)**	***t***	***p***	**Cohen's d (Effect Size)**
Normal	62	51.50 ± 6.24	47.95 ± 7.99	3.965	**< 0.001**	0.504 (Medium)
Benign physiological findings^a^	148	50.96 ± 6.62	45.25 ± 7.73	8.411	**< 0.001**	0.691 (Medium-Large)
Minor structural heart anomalies requiring follow-up^b^	31	51.52 ± 5.16	44.84 ± 7.52	5.335	**< 0.001**	0.958 (Large)

STAI-S, State-Trait Anxiety Inventory State, SD, Standard Deviation, Values in bold indicate statistically significant differences (*p* < 0.05).

a Benign physiological findings: PFO, Includes Patent Foramen Ovale, PPS, tiny Patent Ductus Arteriosus, mild Peripheral Pulmonary Stenosis.

b Minor structural heart anomalies requiring follow-up: ASD, Includes Atrial Septal Defect; VSD, Ventricular Septal Defect; BAV, Bicuspid Aortic Valve; MR, Mitral Regurgitation.

When comparing pre- and post-consultation STAI-S sub-item scores according to ECHO groups, no overall significant change was observed in terms of positive emotional expressions (Reverse items). Although there were minor changes in all groups regarding the items “feeling of relaxation” and “feeling of contentment” after the consultation, these differences were not statistically significant (*p* > 0.05). Regarding the item “ feeling of calmness,” a significant change in post-consultation scores was detected only in the group of benign physiological findings (*p* = 0.014). More significant results were obtained when negative emotional expressions (Direct items) were evaluated. In the item “feeling of tension,” a significant decrease in post-consultation scores was observed in all groups (Normal: *p* < 0.001; Benign physiological findings: *p* < 0.001; Minor structural heart anomalies requiring follow-up: *p* = 0.043). Similarly, significant reductions were observed in all three groups after consultation for the items “feeling upset” and “feeling of worry” (*p* < 0.001 in all comparisons). The results are given in Table 1.

According to the results of the multivariate linear regression analysis conducted to determine the demographic and clinical factors affecting the anxiety levels of parents before and after the examination, the presence of a family history of heart disease in the pre-examination period was found to be the only independent variable with a statistically significant effect on anxiety scores (*β* = 2.129; *p* = 0.031). Parental gender was found to have a borderline significant relationship with pre-examination anxiety levels (*p* = 0.051). In contrast, variables such as parental age, education level, income level, birth order, and number of children in the family did not have a significant effect on pre-examination anxiety (*p* > 0.05). When STAI-S scores were examined after the examination, it was determined that none of the tested demographic or socioeconomic variables had a statistically significant relationship with anxiety level (*p* > 0.05). Full results of the regression model, including *β* coefficients, 95% confidence intervals, and R² values for both time points, are presented in Table 6.

**Table 6:** Multivariate linear regression analysis for factors associated with Pre- and post-examination sTAI-S scores.

**Variable**	***β* (Pre)**	**95% CI (Pre)**	**p (Pre)**	**β (Post)**	**95% CI (Post)**	**p (Post)**
Family history of heart disease (Yes vs. No)	2.129	0.197 to 4.061	**0**.**031**	−0.132	−2.560 to 2.295	0.915
Parental gender (Male vs. Female)	1.793	−0.008 to 3.594	0.051	1.515	−0.749 to 3.779	0.189
Parental age	−0.025	−0.196 to 0.147	0.776	−0.133	−0.349 to 0.082	0.224
Parental education level	1.215	−0.135 to 2.565	0.078	1.245	−0.452 to 2.941	0.150
Family monthly income	0.314	−0.368 to 0.995	0.366	0.721	−0.135 to 1.578	0.098
Birth order of patient	1.523	−1.957 to 5.003	0.390	2.414	−1.960 to 6.788	0.278
Number of children in family	−1.767	−5.207 to 1.674	0.313	−2.682	−7.006 to 1.642	0.223

Pre-examination model: R^2^ = 0.065, Adjusted R^2^ = 0.037, F(7,233) = 2.31, *p* = 0.027.

Post-examination model: R^2^ = 0.034, Adjusted R^2^ = 0.005, F(7,233) = 1.18, *p* = 0.312.

*N* = 241 for both models. Dependent variables: pre- and post-examination STAI-S total scores, respectively. Reference categories: family history = “No”, parental gender = “Female.”.

## Discussion

This study investigated the anxiety levels of parents of children under 24 months of age who were referred to the pediatric cardiology outpatient clinic due to heart murmur, and the effects of expert evaluation and ECHO on this condition. Our study showed a statistically significant and clinically moderate-to-large effect size decrease in parents' STAI-S scores following a detailed examination and ECHO performed by a pediatric cardiologist (from 51.17 to 45.89; *p* < 0.001; d = 0.672). This result strongly parallels findings in the literature indicating that the stress experienced by families whose children have a heart murmur can be alleviated with the intervention of a specialist physician. For example, Akrivopoulou et al. reported a significant decrease in situational anxiety scores after cardiological examination in parents of asymptomatic newborns and infants, and noted that after the interview, the parents felt calmer, less tense, and less anxious (9). Similarly, Bårdsen et al. also showed that evaluation by a pediatric cardiologist significantly reduced parental anxiety (8). In a study conducted in Turkey by Çetin et al., the average STAI-S score of parents referred to cardiology was found to be 41.36, and it was emphasized that hearing a heart murmur caused significant anxiety in parents (1). The relatively higher pre-examination anxiety level (51.17) found in our study compared to the literature may be due to the young age of our patient group (under 24 months) and the feeling of uncertainty associated with this being their first cardiological examination; however, our findings suggest that pediatric cardiology evaluation and echocardiography play an important role in alleviating parental anxiety and reassuring families, as also stated by Ip et al. (6). This non-pharmacological reassurance effect is not limited to cardiology outpatient settings; a recent study by Guo et al. similarly demonstrated that structured pre-procedural guidance significantly reduced parental anxiety and improved satisfaction before transthoracic echocardiography in infants with suspected congenital heart disease (16).

In our study, when patients were grouped according to their ECHO findings (normal echocardiography, benign physiological findings, and minor structural heart anomalies requiring follow-up), a significant decrease in anxiety scores was observed in the parents of all three groups after cardiological evaluation (Table 5; *p* < 0.001). A comparison of the amount of decrease in anxiety scores between the groups (ANOVA) revealed no statistically significant difference (*p* = 0.108). An interesting and somewhat counterintuitive finding of our study is that the effect size of anxiety reduction was largest in the group with minor structural anomalies (Cohen's *d* = 0.958), compared to the benign physiological findings (*d* = 0.691) and the completely normal group (*d* = 0.504). Naturally, one might expect the parents who find out their child's heart is completely normal to experience the most profound relief. This difference cannot be attributed to methodological bias, as the timing of the post-test assessment was standardized across all groups (10–15 minutes post-consultation). Rather, we theorize that this reflects differences in clinical consultation dynamics. When a minor structural anomaly is identified, the cardiologist typically spends more time explaining the specific anatomy and establishes a concrete follow-up plan. This structured medical plan acts as a psychological 'safety net', transforming the parents' catastrophic ‘fear of the unknown’ into a manageable ‘fear of the known’. Conversely, parents of children with normal hearts are discharged without follow-up; the persistence of an audible murmur despite a ‘normal’ diagnosis may paradoxically leave some parents with lingering uncertainty. This observation aligns with recent literature, such as the study by Yang et al., which emphasizes that establishing a clear and structured medical management plan is highly effective in alleviating parental anxiety and excessive worry (17).

In their study examining the effect of ECHO findings on parental anxiety, Akrivopoulou et al. similarly divided patients into three groups and found no significant difference in anxiety scores between the three groups before the examination (9). These findings suggest that when parents first come to the clinic, they experience a high level of uncertainty and stress created simply by the word “murmur,” regardless of the actual anatomical condition of their child's heart. The underlying reasons for this high level of anxiety experienced by parents are well-defined in the literature. In a study by Geggel et al. on parents of children with an innocent heart murmur (Still's murmur), it was shown that families often equated the murmur with a serious heart condition. The study found that 49% of parents were worried about medication use, 41% about sports restrictions, 29% about heart surgery, and 13% about sudden death; furthermore, 19% of mothers blamed themselves for a mistake they made during pregnancy (5). Similarly, in the study by Bårdsen et al., it was reported that 71% of parents had major concerns about murmurs, such as seeing them as a “sign of serious illness” or an “increased risk of future heart disease” (8). The fact that parents come to the clinic with such fears explains why initial anxiety levels remain consistently high, regardless of the findings of the echocardiogram (normal results or minor defects).

On the other hand, although anxiety levels decreased significantly in all three groups after expert consultation and ECHO in the study by Akrivopoulou et al., the anxiety levels of parents in the group with minor structural heart anomalies requiring follow-up on specific short STAI-S items such as “feeling calm,” “being worried,” and “being tense” remained statistically significantly higher than those in the group with normal ECHO (9). However, in our study, the decrease in anxiety among families with benign physiological findings detected on ECHO was similar to that among families with normal ECHO findings, suggesting that effective physician-family communication and the manner in which ECHO findings are conveyed may contribute substantially to parental reassurance. Furthermore, the significant reduction in anxiety following a definitive echocardiographic evaluation even when a structural anomaly is identified, leads us to speculate that parents may experience a psychological transition from the ‘fear of the unknown’ to the ‘fear of the known’. The elimination of uncertainty regarding the child's cardiac health appears to be a major independent driver in alleviating parental distress, complementing the reassuring effect of the physician's communication. To further enhance this reassuring effect, recent literature strongly advocates for the use of modern communication strategies; for instance, Guo et al. (16) and a recent network meta-analysis by Priyadarshini et al. demonstrated that augmenting face-to-face consultations with digital or multimedia educational tools significantly improves parental understanding and reduces procedural anxiety (18) Ip et al. emphasized that incidental and hemodynamically insignificant findings such as PFO or tiny PDA detected during ECHO do not increase anxiety in parents when carefully explained and reassured by the physician (6).

The 87.1% rate of normal or transient functional findings observed in our study is consistent with current literature data showing that approximately 86% of asymptomatic patients referred to pediatric cardiology have no cardiac pathology (2, 3). The fact that the ECHO findings are predominantly benign does not diminish the clinical importance of this assessment; on the contrary, it may provide an effective strategy for what is described in the literature as the ‘morbidity of cardiac nondisease’ (19). Parents may experience high levels of anxiety even based solely on suspicion of a “murmur,” regardless of whether their child's heart is anatomically healthy, leading them to unnecessarily fear surgery, medication, or restrictions. In this context, one potential benefit of ECHO is not only to detect rare major pathologies, but also to alleviate the psychological burden on parents by providing the vast majority of healthy individuals with the greater diagnostic reassurance that there is no structural abnormality in the heart (10, 11). In conclusion, the finding that a minor structural anomaly group (such as ASD or VSD) was identified in 12.9% of our cohort—which, despite not requiring immediate intervention, warrants regular medical follow-up for hemodynamic progression supports the potential clinical value of a comprehensive cardiological evaluation, including echocardiography, in selected patients. While the routine use of ECHO contributes to outpatient clinical costs, our findings suggest that its value extends beyond mere anatomical screening, serving as a powerful and justifiable therapeutic tool for alleviating the psychological morbidity associated with parental anxiety.

In our study, a multivariate linear regression analysis was conducted to determine the demographic and clinical factors affecting parents' anxiety levels before and after examination. The only independent variable that increased anxiety in the pre-examination period was “family history of heart disease” (*β* = 2.129; *p* = 0.031). This finding presents a notable difference from data in the literature. For example, studies by Geggel et al., Bårdsen et al., Akrivopoulou et al., and Çetin et al. in our country have not shown a statistically significant relationship between a family history of heart disease or murmur and parental anxiety (1, 5, 7–9).

This finding suggests that suspected genetic predisposition in the family or previous negative experiences with heart disease are extremely stressful for parents at the time of their child's first visit to the outpatient clinic. On the other hand, the borderline significant relationship (*p* = 0.051) found between pre-examination anxiety level and parental gender in our study is partially consistent with the findings of Bårdsen et al., which showed that mothers exhibited significantly higher anxiety levels compared to fathers (8). This trend is also corroborated by recent findings from Cansaran et al., who demonstrated that maternal anxiety is significantly higher than paternal anxiety in pediatric surgery settings (20). In contrast, other sociodemographic variables such as parental age, education level, income level, birth order, and number of children in the family did not have a significant effect on pre-examination anxiety (*p* > 0.05). Although Bårdsen et al. and Akrivopoulou et al. stated in the literature that low educational level is a significant predictor of increased anxiety, our results support the findings of Geggel et al. and Çetin et al., which indicated that demographic data such as educational level or age are not associated with situational anxiety (1, 5, 7, 8). This indicates that the concern that “the child may have a heart problem” elicits a similar acute stress response in every parent, regardless of socioeconomic and educational status.

A notable finding of our study was that, following expert evaluation and ECHO, none of the tested demographic or socioeconomic variables (including family history of heart disease) were statistically significant in relation to post-examination anxiety levels (*p* > 0.05). This is consistent with Akrivopoulou et al., who observed that post-examination anxiety became independent of education and other factors (7). Thus, while factors such as family history may increase parental stress prior to the examination, a detailed echocardiographic assessment and thorough communication by a pediatric cardiologist appear to have a strong and reassuring effect, effectively offsetting anxiety related to clinical and demographic risk factors. Consequently, clinical workflows should move toward identifying these high-risk families before they even reach the examination room. Specifically, implementing a brief pre-appointment screening checklist for a family history of cardiac disease during the primary care referral stage would allow centers to automatically provide these vulnerable families with targeted educational leaflets or digital information kits. Providing early, structured explanation during the 1-to-2-week waiting period can effectively counter baseline misconceptions and bridge the anxiety-inducing gap prior to the actual consultation. This approach is supported by recent evidence; Cansaran et al. similarly proposed identifying high-risk families for targeted preoperative support in a pediatric surgical setting based on validated anxiety screening (20), while a 2025 network meta-analysis confirmed that structured educational interventions particularly web-based formats produce the largest reductions in parental anxiety prior to pediatric procedures (18).

In response to the limited representation of fathers in previous studies, our cohort included 87 fathers (36.1% of participants), a population that remains underrepresented in studies evaluating parental anxiety associated with childhood heart murmurs and pediatric cardiology referral (5, 8). Interestingly, fathers exhibited slightly higher baseline anxiety scores than mothers and experienced a larger reduction in anxiety following pediatric cardiology consultation. This contrasts with previous literature, which generally reports significantly higher anxiety levels in mothers during pediatric evaluations and procedures (8, 20). Although the study was not specifically designed to investigate gender-related differences in parental anxiety, these findings suggest that paternal responses to diagnostic uncertainty may warrant further investigation in future studies.

## Conclusion

This study revealed that parents of children referred to the pediatric cardiology outpatient clinic due to heart murmurs experienced significantly high levels of anxiety prior to expert evaluation, and that this anxiety appeared to be driven more by clinical uncertainty than by socioeconomic factors. A detailed physical examination by a pediatric cardiologist, echocardiographic imaging, and clear explanation of the findings to the family significantly reduce parental anxiety, regardless of the severity of the cardiac findings in the child. Notably, our regression analysis identified family history of heart disease as an independent predictor of elevated baseline anxiety. Therefore, rather than relying on standard post-examination reassurance, clinical practice should incorporate a systematic family-history screening tool at the referral level. Identifying high-risk families early enables the delivery of pre-appointment counseling and targeted educational materials to mitigate initial distress. In pediatric cardiology practice, recognizing and effectively managing parental anxiety is critical for establishing patient-physician trust and preventing unnecessary restrictions.

## Limitations

Our study has several limitations. First, the absence of a control group limits our ability to distinguish the independent effects of specialist consultation and echocardiography from potential temporal adaptation. Second, we did not collect data on the experience or communication style of referring physicians, which may have influenced baseline parental anxiety. Third, this was a single-center study, and anxiety was assessed only immediately after consultation, precluding evaluation of long-term outcomes. Fourth, our item-level analysis was restricted to the validated 6-item short-form of the STAI-S rather than the full 20 items due to copyright logistics. Although this 6-item short-form has shown high internal consistency in prior validation studies (Cronbach's alpha 0.81–0.85) (9,15), its internal consistency in our cohort was lower (Cronbach's alpha =  0.370 pre-consultation and 0.509 post-consultation), likely reflecting the smaller item pool and cohort-specific response patterns, and this should be considered when interpreting the item-level findings in Table 1. Lastly, psychometric data were obtained from a single accompanying parent rather than dual-parent dyads, which might limit the generalizability of the borderline parental-gender findings. Future multicenter studies with longer follow-up are needed to clarify the relative contributions of specialist counseling and echocardiography to parental reassurance.

## Data Availability

The original contributions presented in the study are included in the article/Supplementary Material, further inquiries can be directed to the corresponding author.
